# Classification and Recognition Method of Non-Cooperative Objects Based on Deep Learning

**DOI:** 10.3390/s24020583

**Published:** 2024-01-17

**Authors:** Zhengjia Wang, Yi Han, Yiwei Zhang, Junhua Hao, Yong Zhang

**Affiliations:** 1 Institute of Precision Acousto-Optic Instrument, School of Instrumentation Science and Engineering, Harbin Institute of Technology, Harbin 150080, China; zhengjiawang@hit.edu.cn (Z.W.); hanxxhit123@163.com (Y.H.); tyutzyw@163.com (Y.Z.); 2 School of Precision Instruments and Opto-Electronics Engineering, Key Lab of Optoelectronic Information Technology (Ministry of Education), and Key Lab of Micro-Opto-Electro-Mechanical Systems (MOEMS) Technology (Ministry of Education), Tianjin University, Tianjin 300072, China; 3 Department of Physics, Tianjin Renai College, Tianjin 301636, China; 4 National Key Laboratory of Science and Technology on Tunable Laser, Harbin Institute of Technology, Harbin 150080, China; 5 Department of Optoelectronic Information Science and Technology, School of Astronautics, Harbin Institute of Technology, Harbin 150080, China

**Keywords:** non-cooperation target, micro-Doppler effect, laser coherence detection, deep learning, classification and recognition

## Abstract

Accurately classifying and identifying non-cooperative targets is paramount for modern space missions. This paper proposes an efficient method for classifying and recognizing non-cooperative targets using deep learning, based on the principles of the micro-Doppler effect and laser coherence detection. The theoretical simulations and experimental verification demonstrate that the accuracy of target classification for different targets can reach 100% after just one round of training. Furthermore, after 10 rounds of training, the accuracy of target recognition for different attitude angles can stabilize at 100%.

## 1. Introduction

With the rapid advancements in aerospace technology, the number of spacecraft in outer space has increased significantly. However, this growing population of spacecraft presents a serious challenge by contributing to the accumulation of space debris and abandoned satellites. This poses a substantial threat to both functioning spacecraft in orbit and the limited resources within the Earth’s orbital space [1]. To ensure astronaut safety and promote the long-term sustainability of the space industry, researchers worldwide are studying measurement techniques specifically designed for non-cooperative targets [2]. These targets include space debris and failed spacecraft that do not respond to traditional tracking and control methods. By developing new measurement techniques for these targets, scientists aim to improve the accuracy and efficiency of monitoring and managing objects in space.

Currently, optical sensors are predominantly used to obtain attitude information from non-cooperative targets [3,4]. Various methods for measuring pose using different sensors exist, such as monocular visual pose measurement, binocular visual posture measurement, three-dimensional laser imaging pose measurement, and microwave radar-based detection methods [3,5,6,7,8]. These methods primarily utilize microwave radar [9,10] and the micro-Doppler effect to collect distinctive features from the target, thus obtaining its attitude information. While this approach offers real-time applicability, the longer wavelength of microwaves makes it difficult to discern the micro-Doppler frequency shift. Consequently, effective micro-Doppler feature extraction for small-sized weakly fretted targets presents a challenge. To overcome this hurdle, the laser coherence detection method [11] was developed. This method detects the micro-Doppler characteristics of laser echo signals to measure the pose parameters of non-cooperative targets, similar to microwave radar detection. The laser coherence detection method is particularly suitable for long-distance detection, allowing for real-time pose parameter measurements with higher accuracy compared to microwave radar [12].

The laser detection-based target identification method primarily consists of three steps aimed at enhancing its effectiveness. Firstly, time–frequency analysis methods are used to convert fretting features into time–frequency spectrograms. Secondly, image processing techniques, such as edge extraction, are applied to acquire the contour information from the time-spectrum map [13]. Lastly, an exclusive algorithm leveraging the target’s prior information is integrated to obtain the required measurement parameters. It is crucial for the surveyor to fully understand the correlation between the time-spectrum change period, frequency shift magnitude, contour trend, and physical characteristics of the target. Additionally, traditional classification recognition methods are limited by manual feature extraction. However, the advancements in deep learning technology, along with the utilization of extensive data, have presented superior classification recognition outcomes. Deep learning technology, supported by high-performance graphics processing units (GPUs), can automatically extract features from vast amounts of data, thus demonstrating robust image classification capabilities.

In this work, we propose a method that integrates the laser coherence detection technique, based on the micro-Doppler effect, with deep learning technology to eliminate the need for manual feature extraction. Furthermore, our proposed approach showcases remarkable precision and accuracy in non-cooperative target classification and identification.

## 2. Modeling Simulation

### 2.1. Micro-Doppler Effect Surface Scattering Model

In real-world environments, different parts of a target often undergo vibrations and rotations, in addition to the primary movement. Examples of such micro-movements include helicopter blade rotation, missile precession, vehicle engine vibration, and pedestrian arm swing. These micro-movements result in frequency modulation of the radar echo signal, which then appears in the frequency sideband near the Doppler frequency shift caused by the main motion of the target. This phenomenon is known as the micro-Doppler effect [14,15,16,17,18,19].

The point scattering model is commonly employed to analyze the micro-Doppler effect. In this model, when the wavelength of the radar signal is much smaller than the size of the target, the target object is perceived as a series of strong scattering points. The radar-measured echo signal can be viewed as the coherent superposition of strongly scattered electromagnetic waves at these local scattering points. Since the laser has a shorter wavelength and higher frequency, the echo intensity of the target is similar for each resolution unit at various distances. Therefore, by applying the concept of plane element segmentation in physical optics, we regard the target surface as consisting of multiple scattering surface elements. In this research, a surface scattering model is utilized for simulating and analyzing micro-Doppler echo signals.

**Figure 1:**
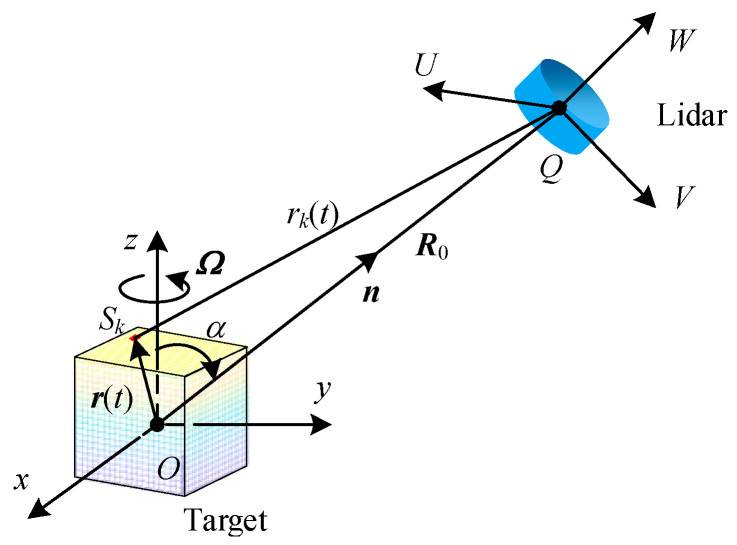
Schematic diagram of a spin cube surface scattering model.

As illustrated in Figure 1, the LIDAR is positioned at the origin *Q* of the radar coordinate system (*U*, *V*, *W*), while the target is located in the local coordinate system (*x*, *y*, *z*) with its centroid at the origin *O* of the coordinate system. For simplicity, this paper does not consider the Doppler shift caused by the target’s translation relative to the radar. Point *Q* is situated in the *yoz* plane of the target’s local coordinate system, and the cubical target is assumed to rotate about the *z*-axis at an angular velocity of *Ω*.

In Figure 1, *S_k_* represents the *k*-th scattering surface element. *α* denotes the target attitude angle—the angle between the unit vector ***n*** for the radar line of sight direction and the spin axis *z*, where ***n*** = [0, sin*α*, cos*α*]*^T^*. Let the initial Euler angle of the rotation target be (0, 0, 0). At the initial moment, the coordinate vector of the scattering surface element *S_k_* is ***r***(0) = [*x*_0_, *y*_0_, *z*_0_]*^T^* and the distance vector between the radar and the target is ***R***_0_. At time *t*, the coordinate vector of the scattering surface element *S_k_* is ***r***(*t*) = [*x*, *y*, *z*]*^T^*. Euler’s rotation matrix ***R****_init_* is a unit array, and the target spin transformation matrix ***R****_spin_* can be expressed as:

(1) $$R_{spin}=\left[\begin{array}{ccc} \cos\Omega t & -\sin\Omega t & 0\\ \sin\Omega t & \cos\Omega t & 0\\ 0 & 0 & 1 \end{array}\right]$$

The coordinate vector ***r***(*t*) of the scattering surface element *S_k_* at *t*-time is given by

(2) $$r(t)=R_{init}R_{spin}r(0)=\left[\begin{array}{ccc} \cos\Omega t & -\sin\Omega t & 0\\ \sin\Omega t & \cos\Omega t & 0\\ 0 & 0 & 1 \end{array}\right]\left[\begin{array}{c} x_{0}\\ y_{0}\\ z_{0} \end{array}\right]$$

Here, *σ_k_* denotes the scattering coefficient of the *k*-th scattering surface element. The target laser echo signal at *t*-time is

(3) $$s(t)=\sum_{k=1}^{K}\sigma_{k}\exp\left[j2\pi f_{0}(t-\frac{2r_{k}(t)}{c})\right],k=1,2,\ldots ,K$$

where *c* is the speed of laser wave, *f*_0_ is the laser carrier frequency. In addition, *r_k_*(*t*) represents the effective distance of the *k*-th scattering surface element *S_k_* relative to radar *Q*, which is written as follows:

(4) $$r_{k}(t)=\left\|R_{0}+r(t)\right\|$$

Ultimately, the micro-Doppler frequency shift caused by the target spin at this point is

(5) $$f_{m-d}=\sum_{k=1}^{K}\left[\frac{2}{\lambda }\frac{dr_{k}(t)}{dt}\right],k=1,2,\ldots ,K$$

### 2.2. Simulation Calculations

The micro-Doppler frequency shown in Equation (5) represents a time-varying signal. Conducting spectral analysis of the echo signal using Fourier transform alone will not capture the frequency variation over time. To address this, our work employs the short-time Fourier transform (STFT), a classical time–frequency analysis method [20,21] which ensures efficient extraction of micro-Doppler features while obtaining the frequency component of the echo signal over time. The STFT involves a mathematical transformation process that uses the window function to intercept the time-domain waveform signal on the basis of the Fourier transform; the corresponding expression is as follows:

(6) $$STFT_{s}(t,\omega )=\int_{-\infty }^{\infty }s(\tau )g^{*}(\tau -t)e^{-j\omega \tau }d\tau =\left\langle s(\tau ),g(\tau -t)e^{j\omega \tau }\right\rangle$$

where “*” symbol denotes the conjugate complex number, *g*(*τ − t*) represents the window function. The window function, which is a real function, is symmetrical about the center *t* = *τ*. In addition, || *g*(*τ*)|| = 1 and || *g*(*τ − t*)*e^jωτ^*|| = 1 must be satisfied. As can be seen from Equation (6), the independent variables of the STFT consists of time *t* and frequency *ω*. In other words, the STFT can display both the frequency composition of the signal and the time distribution information of the frequency. Furthermore, the modulo-square operation of Equation (6) yields the time spectrum *S_s_*(*t*, *ω*)

(7) $$S_{s}(t,\omega )={\left\|STFT_{s}(t,\omega )\right\|}^{2}={\left\|\int_{-\infty }^{\infty }s(\tau )g^{*}(\tau -t)e^{-j\omega \tau }d\tau \right\|}^{2}$$

According to Equation (7), the time spectrum is a constant positive real function that varies with time *t* and frequency *ω*. This time-spectrum result visually shows the time–frequency characteristics of the signal.

**Figure 2:**
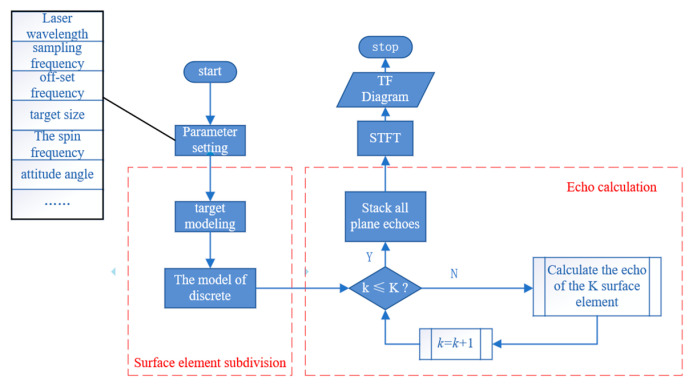
Simulation flowchart.

Three target models were established to approximate the shape of non-cooperative targets: cubes represent satellites in orbit, cones represent rocket debris, and ellipsoids represent space debris. In our simulation, the target model was divided into plane elements, where the level of discretization directly affects the simulation accuracy. By using larger numbers of plane elements, finer simulation results can be achieved, but at the cost of increased computing time and resource consumption. In this work, we divided the surface of the target into 625 scattering surface elements, striking a balance between simulation accuracy and calculation time. Using Equation (3), each surface element echo was calculated and superimposed to obtain the overall micro-Doppler echo signal of the spinning target. The time-spectrum map was then acquired by applying STFT. Figure 2 shows the simulation flowchart, and Table 1 presents the corresponding parameter settings. To identify the target attitude information, we simulated the echo signal using a total of 46 different attitude angles ranging from 0° to 90° in 2° intervals.

**Figure 3:**
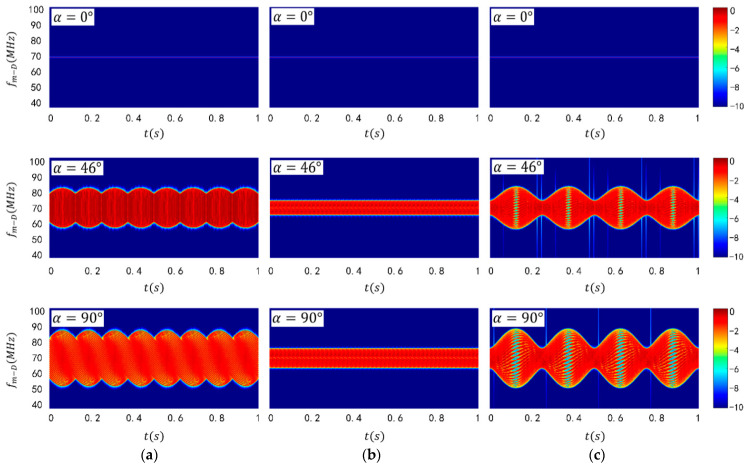
Spectrogram simulation results of (**a**) spinning cubical target, (**b**) spinning conical target and (**c**) spinning ellipsoidal target.

Figure 3a displays a laser micro-Doppler-time spectrogram of a cube-shaped target. It is worth noting that the time-spectrum map obtained through STFT is a three-dimensional spectrum containing information on time, frequency and signal intensity. The horizontal coordinate represents the time dimension of the time–frequency graph, while the vertical coordinate represents the micro-Doppler shift of the laser echo signal, which is the frequency dimension. The third dimension is the signal intensity dimension, depicted using a color-coded time-spectrum diagram. It should be noted that, in this paper, a logarithmic transformation was applied to the intensity dimension of the time-spectrum diagram.

The periodicity over time of the time-spectrum pattern for the spinning cube, cone, and ellipsoidal targets can be observed in Figure 3a–c. When the attitude angle α = 0, the target’s micro-motion lacks a velocity component in the direction of the radar line of sight, thus resulting in a micro-Doppler frequency shift. However, as α = 90°, the target’s spin leads to the maximum velocity component in the direction of the radar’s line of sight and the echo microwave Doppler frequency shift received by the radar reaches its maximum value. In the time-spectrum plot of the spinning cube (see Figure 3a), a twisted rope-like frequency band can be observed. In this work, the cone spins around its central axis, causing the total laser echo signal of the conical target to remain unchanged over time, resulting in a strip pattern with a straight edge for its spectrum. In Figure 3a–c, the time spectrogram also represents a frequency band symmetrically distributed on both sides of 70 MHz. The width of this band is positively correlated with the magnitude of the attitude angle, consistent with the theoretical derivation mentioned earlier. When the attitude angle becomes sufficiently large, significant differences in the micro-Doppler shifts produced by different targets become apparent. It should be noted that for an attitude angle of 0°, the micro-Doppler shift band reduces to a straight line. Difficulties in distinguishing small attitude angles are frequently raised issues with traditional target attitude recognition methods.

## 3. Classification Recognition of Non-Cooperative Targets Based on Deep Convolutional Neural Networks

Traditional non-cooperative object recognition techniques typically employ model-based algorithms to extract the temporal spectrum’s edge. Subsequently, the target parameters can be inversely derived based on the functional relationships within the model. In contrast, deep learning technologies solely require the acquired time–frequency spectrogram for feature extraction, eliminating the need for the manual design of feature parameters. Even for an unknown mathematical model of the target, attitude information recognition can be achieved based on the feature extraction results from the time-spectrum map.

### 3.1. Convolutional Neural Networks

Since AlexNet’s victory in the 2012 ILSVRC competition, deep learning research has experienced a significant surge. Convolutional neural networks (CNNs) have gained considerable attention as a primary structure in deep learning. A CNN is an artificial neural network that operates by forward-propagating input, back-propagating deviation, and updating weights until deviation is sufficiently small. Unlike traditional artificial neural networks (ANNs), CNNs consist of four primary layers: the convolutional layer, the activation layer, the pooling layer, and the fully connected layer [22,23,24,25,26]. The convolutional layer utilizes a convolutional filter to map data from the previous layer to the next, leveraging the receptive field theory and weight sharing to extract local features from the input data. The activation function enhances network feature extraction through nonlinear transformations, typically following the convolutional layer. The pooling layer, also known as the down-sampling layer, is used to reduce data dimensionality. Finally, the fully connected layer classifies the features extracted by the convolutional and pooling layers, functioning as a classification mechanism.

Currently, in the field of image classification and recognition, the most commonly used deep convolutional neural networks are AlexNet, VGG, GoogLeNet, and ResNet. Table 2 offers an overview of the characteristics of each network. Among them, ResNet incorporates a network structure known as a residual block and employs the concept of identity mapping to address the issue of network degradation. Compared to other networks, ResNet has relatively fewer parameters, high accuracy, and performs well in image classification. Therefore, in this work, a deep convolutional neural network based on ResNet was constructed for image classification and recognition, utilizing PyTorch as the deep learning framework. To achieve optimal classification results and avoid excessive computation time, the network depth was set to 32 layers.

**Figure 4:**
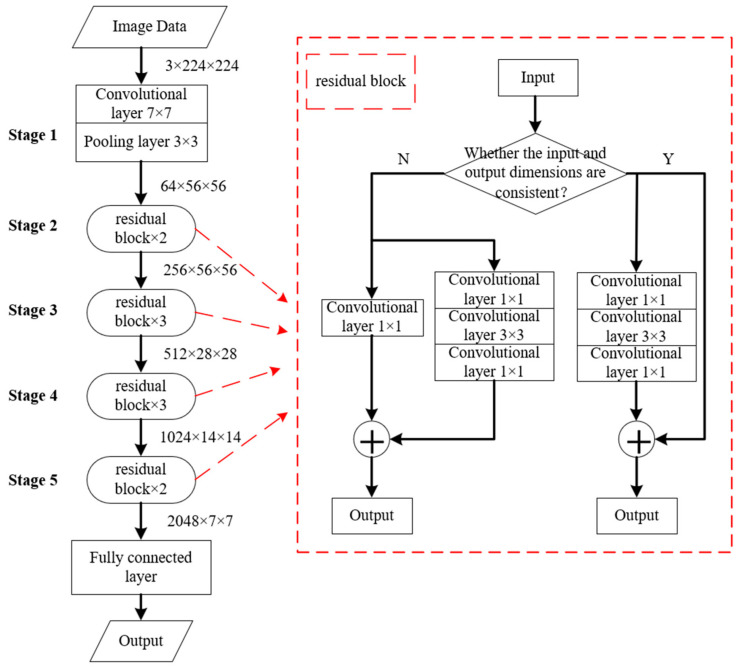
Schematic diagram of the network model structure.

Figure 4 illustrates the model, which consists of five stages. Firstly, the input data undergoes a 7 × 7 convolution followed by 3 × 3 pooling, transforming the image size from 3 × 224 × 224 to 64 × 56 × 56. Secondly, a residual transfer occurs. Downsampling through 1 × 1 convolutional is required to maintain consistent dimensions for identity mapping. The identity map is then added to the result of the convolution, and a new residual block is calculated. This process is iterated, but no downsampling is needed at the new residual block. Stages (3), (4), and (5) are similar to stage (2) with varying numbers of residual block calculations and different data dimensions. Finally, a fully connected layer maps the output data to the sample marker space for image classification.

### 3.2. Classification of Non-Cooperative Targets

To ensure the smooth training and testing of the designed deep convolutional neural network, the initial step involves creating a dataset of simulated time-spectrum maps based on the aforementioned model. This work utilizes two types of datasets: one for target classification, labeled “Dataset_CCE”, and the other for attitude angle recognition, labeled “Dataset_Angle”.

**Figure 5:**
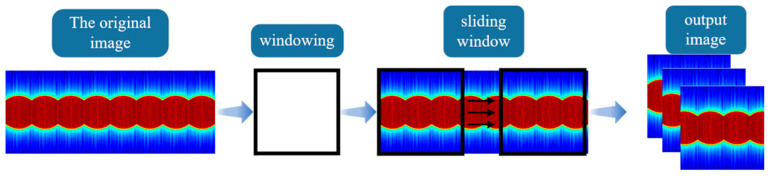
Schematic diagram of sliding window clipping.

A dataset comprising 414 time-spectrum images was simulated for the purpose of the classification of three targets or 46 attitude angles. Each image had a height of 224 pixels and a width of 561 pixels. The slider clipping method was utilized to increase the dataset length for improved training effectiveness. Figure 5 shows the cropping window with dimensions of 224 pixels in height and width. The original image is cropped at every other pixel using the sliding window, resulting in a cropped image size of 224 × 224. This meets the model requirements and expands the dataset length to 1.39518 × 10^5^. To facilitate model training and testing, 60% of the dataset was allocated as the training set, and the remaining 40% was used as the test set.

The parameters involved in the network model training are shown in Table 3. The training effect by the Loss value parameter was mainly investigated. The cross-entropy function CrossEntropyLoss, which is commonly used in the classification problems, is employed as the Loss function here. The cross-entropy function is mainly utilized to determine the approximate degree of the actual output and the expected output. The corresponding Loss calculation formula is given by

(8) $$Loss\left(\overset{̑}{y},y_{class}\right)=-\ln\left(\frac{\exp\left(\overset{̑}{y}[y_{class}]\right)}{\sum_{c=1}^{C}\exp(\overset{̑}{y}\left[c\right])}\right)=\ln\left(\sum_{c=1}^{C}\exp(\overset{̑}{y}\left[c\right])\right)-\overset{̑}{y}[y_{class}]$$

Here, $\overset{̑}{y}$ refers to the network calculation result, *y_class_* is the real class label of one-hot encoding, and *C* represents the number of categories. The lower Loss value from Equation (8) indicates that the two probability distributions are closer. This means the difference between the training result and the true value is smaller.

**Figure 6:**
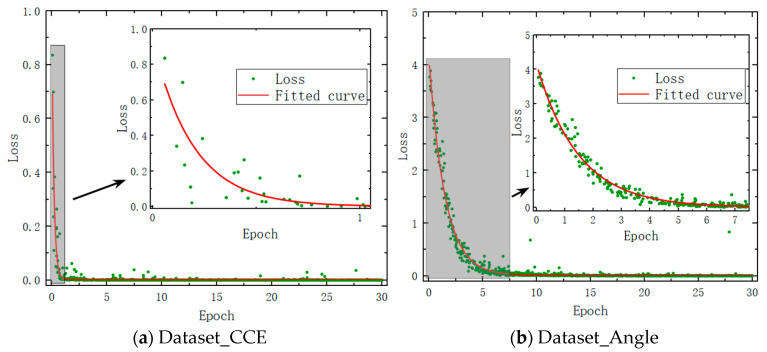
Model training results based on the dataset derived from simulated results.

Figure 6a shows the training outcomes of 30 rounds of training for Dataset_CCE. The loss value exhibits a sharp initial decline, followed by stabilization. The fitted curve (depicted in red) demonstrates that the loss value remains nearly constant at 0 after one round of training. Figure 6b displays the results of Dataset_Angle 30 rounds of training. It can be seen that the loss value also drops more slowly. After seven rounds, the loss value vanishes. Under the same training conditions, Dataset_CCE has fewer categories. It means that a single category is trained with a larger volume of data, and the extraction of feature points is more adequate. Accordingly, Dataset_CCE demonstrates superior training effectiveness and a faster convergence speed.

Testing is a key step in validating the effectiveness of this method. In this paper, the training effect of the network model is mainly evaluated by accuracy. The corresponding calculation formula is written as

(9) $$Accuracy=\frac{\sum_{c=1}^{C}S_{c}}{L_{test}}$$

Here, *C* represents the total number of categories. In this work, *C* = 3 and 46 for Dataset_CCE and Dataset_Angle, respectively. *S_c_* denotes the total number of data classifications for class c that are correct, and *L_test_* is the test set length.

**Figure 7:**
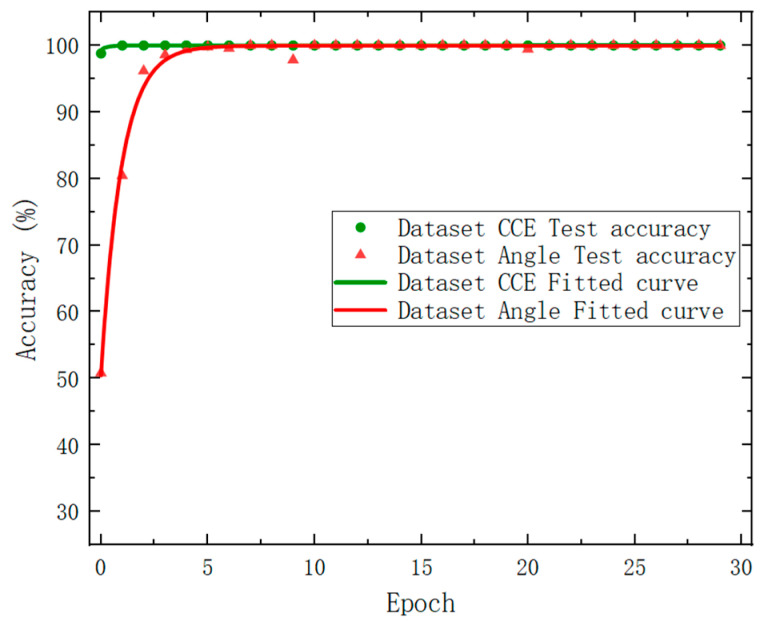
Model test results based on the dataset derived from simulated results.

The test results are shown in Figure 7, where the green circular data points represent the correct test rate of Dataset_CCE, and the green curve is the nonlinear fit result of its distribution trend. It was found that the overall test effect for the Dataset_CCE test set was superior. After round 1, the test accuracy rate stabilized at approximately 100%. The red triangle data points in the plot denote the corresponding test accuracy of the Dataset_Angle, and the red curve is the nonlinear fit result of its distribution trend. It can be seen that after seven rounds, the accuracy rate basically tended towards 100%. The Accuracy and Loss are in opposite trends during training, which is consistent with theoretical analysis.

## 4. Experimental Validation

In the field of laser detection, two primary methods are utilized: incoherent detection and coherent detection. Incoherent detection primarily measures the power change in the echo signal to acquire target information. While the device used for incoherent detection is simple, it encounters challenges in obtaining frequency information. On the other hand, coherent detection methods, although more complex, offer numerous advantages. They not only detect light intensity but also have the capability to measure frequency and phase. Coherent detection is characterized by high sensitivity, large photoelectric conversion gain, and excellent filtering performance [27]. To effectively acquire the micro-Doppler frequency shift information of the echo signal, this research implements the laser coherence detection method for data acquisition.

### 4.1. Experimental Data Acquisition

**Figure 8:**
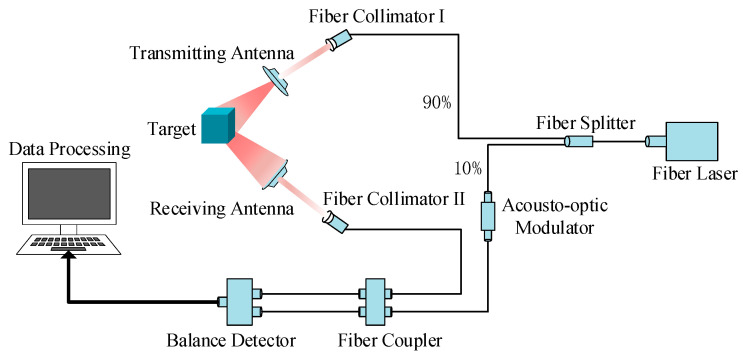
Schematic diagram of the experimental apparatus.

Figure 8 displays a schematic diagram of the experimental setup. The working process is outlined as follows: A fiber laser generates a 1064 nm wavelength laser (Shanghai Han Yu Fiber Laser Co., Ltd., Shanghai, China), which is able to achieve high micro-motion spectral resolution and longer working distances. Utilizing a 1064 nm fiber laser allows a KHz-level linewidth to be achieved through a seed source laser, and subsequently substantial laser output power through fiber amplification to be attained. In addition, atmospheric scattering is a crucial factor to consider for laser detection. The atmosphere can have absorption and scattering effects on the laser. On one hand, the 1064 nm laser operates within the atmospheric transmission window, resulting in minimal atmospheric absorption. On the other hand, compared to shorter wavelength lasers, the longer wavelength of the 1064 nm laser results in less atmospheric scattering, further justifying our choice of the 1064 nm laser. The laser beam is then split into two parts after passing through a fiber splitter. Ten percent of the laser undergoes a carrier frequency increment of 70 MHz through an acousto-optic modulator, serving as the local vibration light for coherence. The remaining 90% of the laser beam is directed towards the target to be measured via the emitting optical system, and subsequently, the scattered echo signal is collected by the receiving optical system. The local oscillation and echo signals are combined using an optical coupler. It should be noted that in this type of detection, there is an optimal value for the local oscillator light, corresponding to the highest signal-to-noise ratio in the system. Deviating from this optimal value reduces the signal-to-noise ratio. According to our tests, a 10/90 splitting ratio is the optimal ratio. The resulting differential frequency signal is converted into an electrical signal output by a balanced photodetector. The computer then performs data processing to obtain the time spectrogram of the target. Finally, deep learning technology enables the classification and identification of the goal’s time-spectrum map. The experimental parameters are detailed in Table 4.

**Figure 9:**
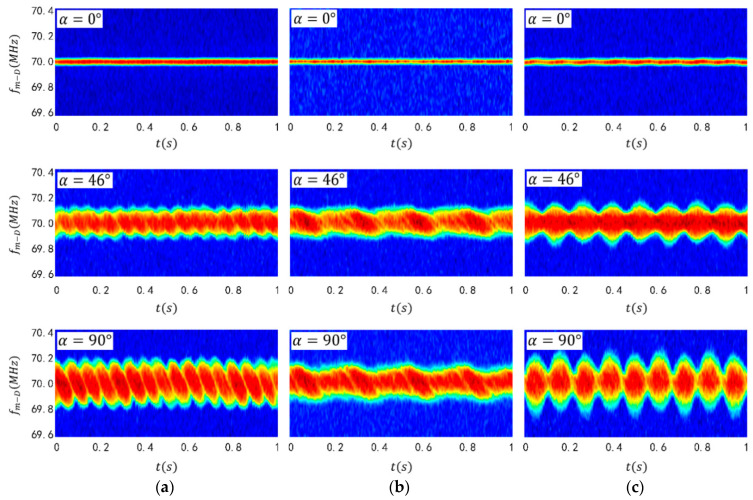
Spectrogram experiment results of (**a**) cubical target, (**b**) conical target and (**c**) ellipsoidal target.

The experiment’s spectrograms are shown in Figure 9. As seen in the spectrogram of the cubical target (Figure 9a), the band width increases with the attitude angle. The spectrogram presents itself as a symmetrical twisted rope band of approximately 70 MHz, consistent with our simulation results. Note that the experimental target size was small, in the millimeter range, resulting in a small target spin radius. The maximum micro-Doppler shift in the spectrogram is around 0.2 MHz, smaller than the simulation. Figure 9b displays the spectrogram experiment results of the conical target. It is noticeably different from the simulation results presented in Figure 3b. The spectrum edges are not perfectly straight due to the shift in the spin target rotation axis during the experiment. Additionally, the target’s rotation precession causes periodic concave-convex variations at the edge of the time spectrum. Figure 9c shows the spectrogram experiment results of the ellipsoidal target, which largely align with the simulation. However, the scattering intensity at the target edge, corresponding to the maximum micro-Doppler shift, is weak in the experimental spectrogram, resulting in poor contour clarity.

### 4.2. Experimental Results and Discussion

In the experiment, we collected a total of 138 time-spectrum maps of three targets at 46 different attitude angles. To expand the dataset and ensure that the image size met the requirements for training and testing the network model, we also used the sliding window cropping method to process the data. The experimental dataset was divided into two parts: Dataset_CCE for target classification and Dataset_Angle for pose angle recognition. The total length of the dataset was 1.08468 × 10^5^. Similar to the simulation described earlier, 60% of the data were used as the training set for model training, while the remaining 40% were used as the test set to evaluate the training effect.

**Figure 10:**
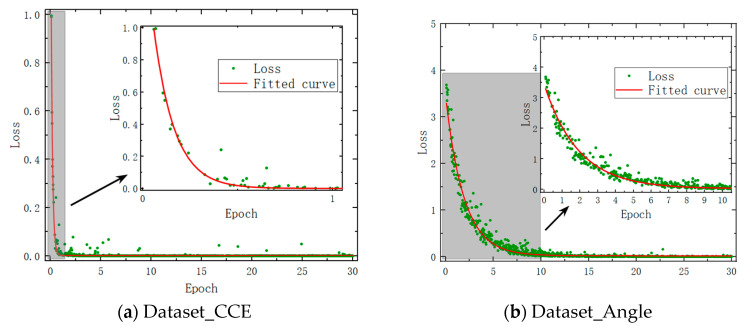
Experimental dataset model training results.

According to our analysis, three factors negatively impacted the data obtained from the experiment: (1) The signal-to-noise ratio of the time–frequency plots obtained from the experiment was lower; (2) The motor driving the rotation of the target model exhibited a slight vibration during operation; (3) The size of the target model used in the experiment was in the millimeter range. This resulted in narrower sidebands in the echo signal’s time–frequency plots and smaller Doppler frequency shifts. As a result, the spectral resolution of the time–frequency plots obtained from the experiment was lower. The aforementioned reasons led to more uncertainty in the experimentally obtained time–frequency plots compared to those obtained from simulations. This uncertainty increased the difficulty of fitting with deep learning and affected the fitting speed of the experimental data. The training results are illustrated in Figure 10. Figure 10a shows the results of 30 rounds of training for Dataset_CCE. The maximum Loss value at the beginning was 0.99, which quickly dropped to 0 after one round of training, indicating fast convergence. Figure 10b displays the results of 30 rounds of training for Dataset_Angle. The maximum Loss value at the beginning was 3.69. It can be observed that after 10 rounds of training, the Loss value basically disappeared. Based on the simulated dataset, only seven rounds are needed (see Figure 6b). The Dataset_Angle, which has more categories, demonstrated a slower convergence speed and larger initial training output Loss compared to Dataset_CCE. However, with only three categories, Dataset_Angle showed a better training effect. This observation is consistent with the qualitative results obtained from the simulations.

**Figure 11:**
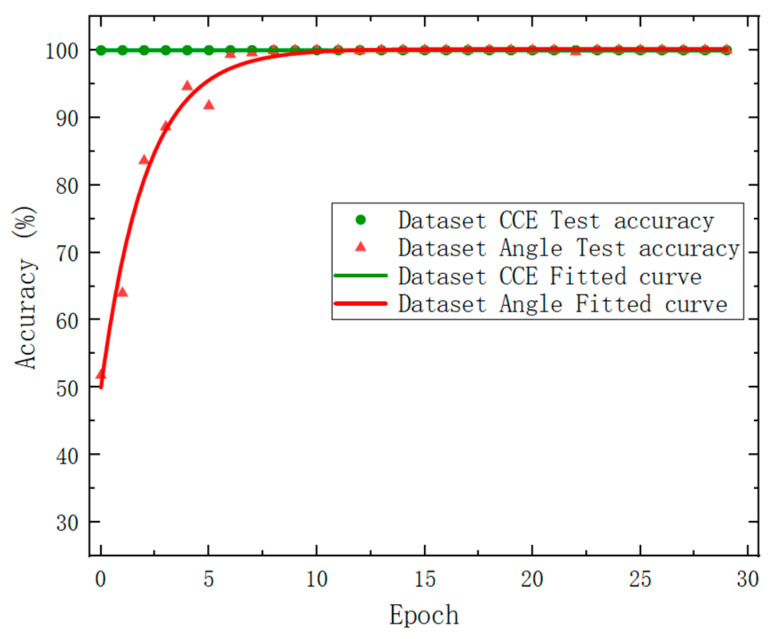
Network model test results.

Figure 11 shows the test results of Dataset_CCE as represented by green circular data points, characterized by a green fitting curve. Similarly, the test results of Dataset_Angle are represented by red triangle data points and featured by a red fitting curve. The target classification dataset exhibited high accuracy of 99.97% even in the first round, and the overall accuracy rate of 100% was maintained throughout. However, the attitude angle recognition dataset had a lower accuracy rate than the other dataset in the early rounds, with a first-round accuracy rate of just 52.04%. Nevertheless, after 10 rounds, the accuracy rate was able to reach and maintain at 100%. Note that only seven rounds are needed based on the simulated dataset (see Figure 7). To sum up, although the fitting speed of the experimental data was slightly slower than that of the simulated data, it can be seen that, after iteration, our deep learning network achieved a similar accuracy and loss function value to the simulated data. This fully demonstrates the effectiveness and robustness of this method in identifying the types and attitude angles of non-cooperative targets.

## 5. Conclusions

This work proposes a method using deep learning technology to identify non-cooperative targets with high accuracy. The proposed method is based on the micro-Doppler effect with laser coherence detection serving as the measurement approach. In the simulation, the classification and recognition accuracy reached 100%. Furthermore, an experimental platform was built using laser coherence detection, and the experimental results demonstrated that this method effectively classifies and recognizes non-cooperative targets, achieving an accuracy rate as high as 100%. The simulation and experiment validate the effectiveness of our proposed method for the classification and recognition of non-cooperative targets, and its great potential in the field of non-cooperative target measurement. This method holds promise to play a pivotal role in ensuring high accuracy in identifying non-cooperative targets by machines.

## Figures and Tables

**Table 1:** Simulation parameters of spin targets’ laser micro-Doppler echo signals.

Parameter Name	Value
The side length of the cube	0.5 m
Cone base radius/cone top height	0.5 m/1.5 m
Ellipsoid short/medium/long axis	0.5 m/0.5 m/1.5 m
Spin frequency	1 Hz, 2 Hz, 3 Hz
Attitude angle *α*	0°, 2°, 4°, …, 90°
Window function	Hanning
Laser wavelength	1064 nm
Bias frequency	70 MHz
Sampling frequency	200 MHz
Simulation duration	1 s
The number of face elements	625
Window length	2 × 10^4^ points

**Table 2:** Comparison of characteristics of deep convolutional neural networks.

	AlexNet	VGG	GoogLeNet	ResNet
Birth time	2012	2014	2014	2015
Number of layers	8	19	22	152
Error rate of Top5	16.4%	7.3%	6.7%	3.57%
Memory consumed	High	High	Low	Lower

**Table 3:** Training and test parameters’ table.

Parameter Name	Value	Parameter Name	Value
Equipment	cuda	Learning rate	0.001
Loss function	CrossEntropyLoss	Epoch	30
Optimizer	SGD	Batch size	16
Training set length	8.3711 × 10^4^	Number of categories	3/46
Test set length	5.5807 × 10^4^	—	—

**Table 4:** The experiment involves a parameter table.

Parameter Name	Value
The side length of the cube	6 mm
Cone base radius/cone top height	4 mm/12 mm
Ellipsoid short/medium/long axis	4 mm/4 mm/12 mm
Drive voltage	10 V
Attitude angle *α*	0°, 2°, 4°, …, 90°
Laser wavelength	1064 nm
Bias frequency	70 MHz
Sampling frequency	200 MHz
Emits laser power	300 mW
Detection distance	80 m

## Data Availability

Data are contained within the article.
